# Cities through the Prism of People’s Spending Behavior

**DOI:** 10.1371/journal.pone.0146291

**Published:** 2016-02-05

**Authors:** Stanislav Sobolevsky, Izabela Sitko, Remi Tachet des Combes, Bartosz Hawelka, Juan Murillo Arias, Carlo Ratti

**Affiliations:** 1 Center For Urban Science And Progress, New York University, Brooklyn, New York, United States of America; 2 Senseable City Lab, Massachusetts Institute of Technology, Cambridge, Massachusetts, United States of America; 3 Department of Geoinformatics - Z_GIS, University of Salzburg, Salzburg, Austria; 4 Data & Analytics, BBVA, Madrid, Spain; University of Namur, BELGIUM

## Abstract

Scientific studies of society increasingly rely on digital traces produced by various aspects of human activity. In this paper, we exploit a relatively unexplored source of data–anonymized records of bank card transactions collected in Spain by a big European bank, and propose a new classification scheme of cities based on the economic behavior of their residents. First, we study how individual spending behavior is qualitatively and quantitatively affected by various factors such as customer’s age, gender, and size of his/her home city. We show that, similar to other socioeconomic urban quantities, individual spending activity exhibits a statistically significant superlinear scaling with city size. With respect to the general trends, we quantify the distinctive signature of each city in terms of residents’ spending behavior, independently from the effects of scale and demographic heterogeneity. Based on the comparison of city signatures, we build a novel classification of cities across Spain in three categories. That classification exhibits a substantial stability over different city definitions and connects with a meaningful socioeconomic interpretation. Furthermore, it corresponds with the ability of cities to attract foreign visitors, which is a particularly remarkable finding given that the classification was based exclusively on the behavioral patterns of city residents. This highlights the far-reaching applicability of the presented classification approach and its ability to discover patterns that go beyond the quantities directly involved in it.

## Introduction

Laws and regularities in human behavior have been the subject of intense research for several decades. In the age of ubiquitous digital media, different aspects of human activity are being increasingly analyzed by means of their digital footprints, such as mobile call records [1–5], vehicle GPS traces [6], social media activity [7–10], or smart card usage [11, 18]. The wide popularity of debit and credit cards, which are increasingly replacing cash spending, suggests the appearance of yet another source of valuable information for scholars across a wide range of disciplines. Extensive transactions datasets, collected by banks and other providers of payment systems, allow a glimpse into the daily activities of large numbers of individuals. While the spatiotemporal granularity of this data may be sparser compared to previously explored sources, such as call detail records (CDRs), incorporated contextual information such as spending category, amount and place enables the analysis not only of movement patterns but also of the semantic and context of human activity. Furthermore, demographic profiles of bank customers provide an additional important layer to explain such activity.

In urban studies, the analysis of aggregated bank card data may provide novel insights in the description and comparison of the economic dimension of cities, in a way that goes beyond other types of digital records used in the past to investigate urban [12] and regional structure [13], land use [14, 15], mobility [16, 17], or well-being [18]. Adequate and reliable metrics are of a primary importance for a city management, especially in the context of the increasingly competitive global economy [19]. In this paper, we propose to look at the socioeconomic conditions of urban areas through the lens of their inhabitants spending activity, based on a dataset that ensures uniform and up-to-date description of a multitude of cities and an approach that compensates for their various sizes and characteristics.

Previous studies of individual economic activity were mostly based on field studies [20], questionnaires [21], and surveys [22]. Direct analysis of individual bank card records has not been extensive so far. Card transactions data is highly sensitive, includes a lot of private information and requires extensive efforts to anonymize it appropriately. Therefore, its access has been so far highly restricted. Hitherto applications have mostly been focused on the card system itself [23, 24], rather than on the associated human behavior. More recently, Krumme et al. [25] employed this new type of data to uncover the predictability of spending choices, and their relationship to wealth. A comparable dataset of retail market transactions, treated as a complex system encompassing customers and the products they purchased, was used to uncover the hierarchy of customers’ needs and a propensity for shopping-driven travels [26]. The analysis of bank card data was also carried out in the field of regional delineation [27], human mobility [28], as well as the assessment of city attractiveness for different groups of customers [29, 30]. However, a comprehensive analysis of human spending behavior in cities has yet to be performed—such is the scope of the present study.

### Factors affecting individual economic activity of city residents

Shopping patterns of individuals were found to depend on demographic factors such as gender, age, education, occupation, or income [22, 28, 31, 32]. However, their exact impact on the propensity for bank card usage is reported differently across various studies. Regarding gender, some results indicate an increased likelihood of bank cards usage among women [33, 34], while others point to their preference for checks over cash or cards [35]. Women are further reported to spend more money and perform a higher number of transactions than men [28]. Age is reported to either lower the probability of card usage [34], have no significant effect [35], decrease or increase spending activity depending on gender [28]. Given those discrepancies, as well as the possibly different characters of the analyzed cities, we begin this study with a detailed analysis of the impact of demography on individual bank card transactions and then normalize the aggregated spending profiles of cities accordingly.

Another important context for human economic activity is its geographic location. In the case of urban customers, this notably concerns the city of residence and in particular its size, quantified via its population. Due to agglomeration effects and intensified human interactions, a variety of urban processes have been shown to vary with the number of inhabitants in the form of scaling laws [36–39]. While urban infrastructure dimensions (e.g., total road surface) reveal a sublinear relation to city size, socioeconomic quantities (e.g., gross metropolitan product, crime rate, patenting, and human interactions) usually increase in a superlinear manner [40]. One can expect that human spending in urban areas holds similar property, which is one of the hypothesis that will be tested below.

### Organization of the paper

In this paper, individual purchase activity measured using bank card records is explored in order to discover collective patterns of economic activity. Based on those, we propose a novel approach to compare and classify Spanish cities in terms of the spending behavior of their inhabitants. The results are presented in three main sections.

We start by asking a broad range of questions regarding the fundamental factors that impact economic conduct. At first, we reveal the ways that demographic factors, such as age and gender, influence five representative quantities of individual bank card spending in Spain during the year 2011. That step allows us to look beyond the impact of the demographic heterogeneity of cities. In the next part, we then investigate the impact of city of residence, studying whether people from different places tend to spend their money in different ways. In line with previous studies on the scaling laws governing urban quantities, we examine the impact of city size on the economic activity of its inhabitants. The discovered trends give a good reference point for the expected collective behavior in a city of a given size. However, each particular city demonstrates a unique performance. Therefore in the final section, following the approach of [41], we propose an index that measures the relative performance of cities based on the deviations of spending parameters from the general trends, indirect effects of population specificity taken into account. Such index allows for the comparison of cities of different population sizes and forms the basis for a novel scale-free classification of Spanish cities based on the economic behavior of the residents. The classification is given a thorough geographic and socioeconomic interpretation, revealing meaningful patterns and different characters of urban areas in Spain. We end the paper with the summary of its main findings and conclusions.

## Materials and Methods

### Data set of bank card transactions

Our study relies on the complete set of bank card transactions (both debit and credit) performed by the Spanish customers of Banco Bilbao Vizcaya Argentaria (BBVA) within the country in 2011. The total number of active customers reaches around 4.5 M, who executed more than 178 M transactions, with a cumulative spending exceeding 10.3 billion euro. Due to the sensitive nature of bank records, they were anonymized by BBVA prior to sharing, in accordance to all local privacy protection laws and regulations. Randomly generated IDs of customers are connected with certain demographic characteristics and an indication of a residence at the level of zip code, further aggregated into coarser spatial units. Each transaction is denoted with its value, a time stamp, the location of the point of sale where it was performed, and the business category it belonged to. The business classification includes 76 categories such as restaurants, gas stations, supermarkets or travels. In order to compensate for the inhomogeneous penetration of BBVA on the individual banking market in Spain, we normalize—in the first section concerning demography—the activity of customers by the BBVA market share in their respective residence location (provided by the bank). Then, using conclusions from that first section, we design a second normalization procedure to take into account demographic differences between cities. The raw data set is protected by a nondisclosure agreement and is not publicly available. However, certain aggregated and normalized data can be obtained from http://senseable.mit.edu/BBVA/LUZ_CON_FUA_spendingSignatures.xlsx for the purpose of findings validation. For the three types of Spanish cities introduced below, the dataset contains their names, population counts, location, normalized performance characteristics introduced in the present paper (spending activity, average transaction amount, spending diversity, local and distant mobility, attractiveness for foreign visitors), normalized statistical characteristics (unemployment, household income, GDP) as well as the cities’ membership in the clusters, constructed in the paper. Additional aggregated and anonymized data could be shared (subject to the bank’s approval and entering an appropriate non-disclosure agreement) upon request to be addressed to María Hernández Rubio at maria.hernandezr@bbvadata.com.

### Major characteristics of customers’ spending behavior

In order to characterize the spending behavior of customers, we consider five basic parameters of bank card usage. Three of them are related to the economic dimension of transactions:

- The activity of each customer, defined as the total number of transactions performed during a year
- The average value of a single transaction
- The spending diversity, measured as the inverse of the Herfindahl-Hirschman Index for business categories visited by a given customer in 2011 (i.e. the inverse of the sum of the squared visit frequencies to each business category).

Additionally, we introduce two characteristics of customers’ mobility, following evidence connecting human mobility and spending behavior provided by [26] and [28]:

- Distant mobility, measured as the percentage of transactions executed over 200 km from home
- Local mobility, measured as the average distance between the customer’s home location and the retail points (calculated based on transactions made within 100 km from home)

Correct computation of four of the aforementioned quantities (all but activity) requires the customer to be using his bank card frequently enough (e.g., there is no point in measuring the spending diversity or mobility of someone who used a card only a couple of times). In the further analysis, we thus only consider customers who performed at least 50 transactions in 2011 (which gives an average close to one transaction per week). Moreover, we restrict the analysis to customers active during the entire year i.e., those who performed transactions during at least nine different months. All five characteristics of spending behavior are further considered at the city scale–as an average value across the activity of residents.

### Three levels of city definition

For a spatial definition of Spanish cities, we test three different types of units. The coarser city level consists of 24 Large Urban Zones (LUZ) as defined by the European Urban Audit Survey [42]. The intermediate level concerns 211 Conurbations (CON) identified within the AUDES project (Aŕeas Urbanas de Espanã) [43], further limited to 205 due to data sparsity for some of the smaller towns. For the finer spatial scale, we aggregate Administrative Cities of Urban Audit into 40 Functional Urban Areas (FUA), so as to reflect metropolitan regions in agreement with the Study on Urban Functions of the European Spatial Planning Observation Network (ESPON) [44]. Population and socioeconomic statistics for LUZ and FUA levels were obtained from Eurostat [42] and the Spanish National Statistics Institute [45]. Population figures for the CON level comes from the AUDES project [43].

## Impact of demography on customer behavior

Among the primary factors that affect human economic behavior are age and gender [22, 31, 32]. Important observations on the influence of sociodemographic characteristics on human spending habits were already introduced by [28]. In this section, we verify and extend those findings to our five measures of bank card usage (i.e., customer’s activity, average value of a transaction, diversity of transactions, as well as distant and local mobility). Good understanding of the demographic conditioning of those parameters is a crucial pre-processing step for our analysis: a comparison of the aggregated spending patterns in demographically diverse cities across Spain.

**Fig 1:**
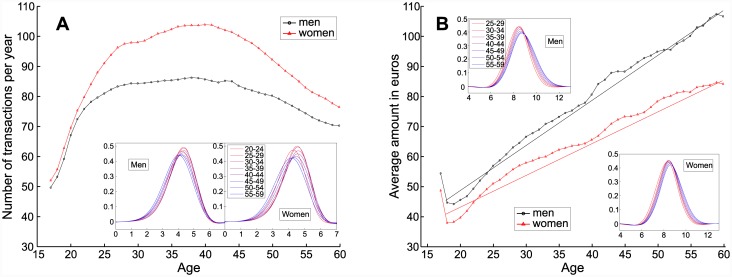
Impact of age and gender on selected parameters of customers’ spending behavior. A: Average number of transactions per year. B: Average value of a single purchase. Insets: Logarithmic distributions for both genders and different age groups.

**Fig 2:**
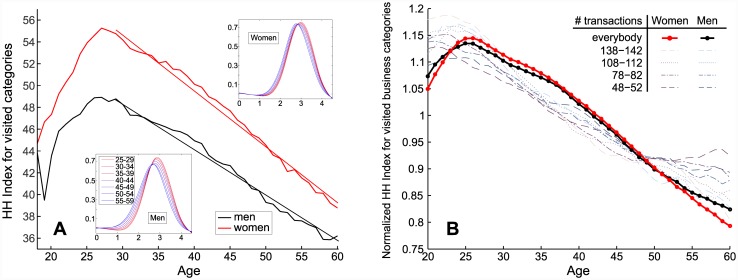
Impact of age and gender on customers’ spending diversity. A: Average spending diversity against age for men and women. B: Normalized Herfindahl-Hirschman Index of visited business categories for the customers with different levels of spending activity.

**Fig 3:**
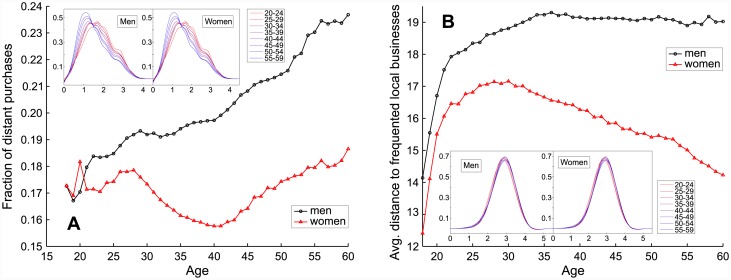
Impact of age and gender on customer mobility. A: Frequency of distant travels. B: Average distance to home of local transactions. Insets: Logarithmic distributions for both genders and different age groups.

We present results for respective parameters in Figs 1–3. From a global perspective, one can observe that even though trends for both genders are substantially different quantitatively, in most cases, they exhibit remarkable similarities in their shape from a qualitative viewpoint. For instance, the number of transactions is usually higher for women, while the average value per transaction is higher for men, who seem to concentrate their economic activity more than women. Also, while the spending diversity of women customers is higher, their mobility is substantially lower on average. Nevertheless, the tendency for both men and women, as well as the important age thresholds where these tendencies change, remain strikingly similar. Looking beyond the simple average values of each characteristic we also observe the steady and continuous impact of customers’ demographics on the shape of parameters’ distributions. Let us now take a closer look at the respective parameters.

### Customer activity and average amount per transaction

In Fig 1A, the average number of transactions per year is plotted against age, for both men (in black) and women (in red). We also aggregated the data into age groups and plotted the distribution of the number of transactions (in log) for five-year brackets. One can see that customer activity increases rapidly between 18 and 30 years old, as expected with the entry into the workforce. After reaching its peak, it remains more or less constant for both genders till 40 years, and then steadily decreases. From an economic point of view, it thus appears that people are most active during their 30s. Moreover, comparing the two curves shows that women make every year on average 15.8% more transactions; that number goes up to 20.2% when considering customers in the 25–45 age group. Peak period of activity and a higher number of transactions executed by women agrees with the observations introduced in [28].

After the number of transactions, let us focus on their average amount (Fig 1B). It is quite remarkable that this quantity grows with the customers’ age in a nearly linear way, doubling between the youngest (18 years old) and the oldest (60 years old) customers. Intuitively, this effect could be connected to the ability of older people to spend more or to buy more expensive goods (earning more money as their career develops). Nevertheless, it does not go in line with the pattern displayed by the total amount of spending, a quick increase till 40 years old and then fewer variations (the actual graph can be found in S1 Fig). Thus, it seems more natural to explain the steady increase of the average purchase value by a habit of concentrating purchases, making fewer transactions but buying more each time.

As to gender differences, the average amount per transaction is smaller for women, who appear to spend more often, but for smaller values. These two statements might be related to the fact that between the age of 20 and 60 years old, only 75% of Spanish women compared to 89% of men are professionally active [45]. This indicates that, although the situation is evolving (the percentage of working women was only 65% in 2005), women remain more involved in domestic tasks, in particular the essential shopping, using their bank card more frequently and for smaller amounts.

### Spending diversity

Given the data at hand, we are also able to analyze the diversity of human economic behaviors based on a variety of places where people spend their money. In Fig 2, we correlate the inverse of the Herfindahl-Hirschman Index for the number of visited business categories with customers’ age and gender. We considered the inverted index to make it an increasing function of diversity. After a rapid increase until the age of 27–28, the diversity of spending declines steadily in a linear way, showing that, while people spend more money growing old, they also tend to spend it in fewer types of businesses. This abrupt change in the trend occurring in the late 20s well matches the time one founds his/her family [46].

As far as gender is concerned, we notice that women tend to visit a larger number of business categories. This raises a question, however. In Fig 2A, the average was taken on every customer of a given age regardless of their total number of transactions, and we have seen that age greatly impacts the transaction activity (which in turn could impact diversity). To ensure that diversity measures are not biased by different activity levels at different ages, we group customers according to their level of activity (from 50 +/- 2 to 140 +/- 2) and plot in Fig 2B the normalized number of visited business categories for each group. We also plot the same quantity for the entire set of customers (the thick black and red lines). The graphs exhibit the pattern already seen in Fig 2A, which confirms our previous conclusions.

### Customer’s mobility

While the home location of a customer is irrelevant to the aforementioned considerations, it becomes essential when studying human mobility. In the data, for each anonymized customer ID is given a zip code of the residency address. However, as far as each individual customer is concerned, the exactness of this formal zip code is questionable (e.g., students registered at their parents’ home, people moving and not informing their bank, etc.). To get rid of that bias, we compute the fraction of transactions that took place in the daily accessible neighborhood of the reported home zip code and discard all customers for which said value is smaller than 60%. They represent around 18% of customers.

In Fig 3, we plot the two parameters of customers’ mobility (percentage of transactions performed more than 200 km away from home and average distance traveled to businesses less than 100 km from home) against age for both genders. The distant mobility (Fig 3A) is the first quantity displaying a big difference in trends between men and women under 40 years old. While for men, the fraction of distant purchases increases with age in a roughly linear way, distant mobility of women first stagnates, then decreases until 40 years old, and finally starts to increase similarly to the curve observed for men. In a parallel way to the analysis of activity, this difference can be connected with a societal explanation. The average age for childbearing in Spain is 29.8 years old [46], which strikingly corresponds to the change in the curve evolution. Regarding local mobility, an interesting pattern can be identified in Fig 3B. While local mobility of men remains nearly stable after 25–30 years old, with only a very slight tendency to decrease with age, women exhibit significant and stable decrease of the average distance to visited local retailers. They tend to shop closer to their home when growing older, which, together with the overall shorter distance of local purchases, well agrees with the findings of [28]. Additionally, from the propensity for shopping-driven travels perspective (proven to increase with the sophistication level of purchased products [26]), we can confirm here as well that women are more involved in basic shopping needs, as already suggested in the section dedicated to the average value of purchases.

## Bigger cities boost up spending activity

It is well established that living in a bigger city boosts up many aspects of human life: intensity of interactions [39], creativity [41], economic efficiency measured in GDP [40], as well as certain negative aspects such as crime [41]. In the following section, we examine whether this property holds true for the individual economic activity of city residents. To do so, the average values of five bank card usage characteristics are quantified and their dependence on city size (expressed in terms of population) is analyzed. As the urban scaling laws were found sensitive to the selection of city boundaries [47], we test and compare three levels of city definition, namely, Large Urban Zones (LUZ), Functional Urban Areas (FUA), and Conurbations (CON).

In the previous section, we prove individual economic behaviors to depend on customers’ demography. It thus appears necessary to take into account possible variations of demographic profiles between different cities. As a matter of fact, age and gender vary quite significantly from one city to another. Among the 24 LUZ, for instance, the fraction of male customers varies between 47.5% and 51%, the average customer age goes from 41 to 48 years old, and the respective fractions of different age groups change up to a factor of 1.7. In order to correct for that demographic heterogeneity, we normalize each of the city characteristics by their expected value (computed using the demographic composition of the city and the average parameters estimated on the entire set of customers; for more details, see S1 Text).

**Fig 4:**
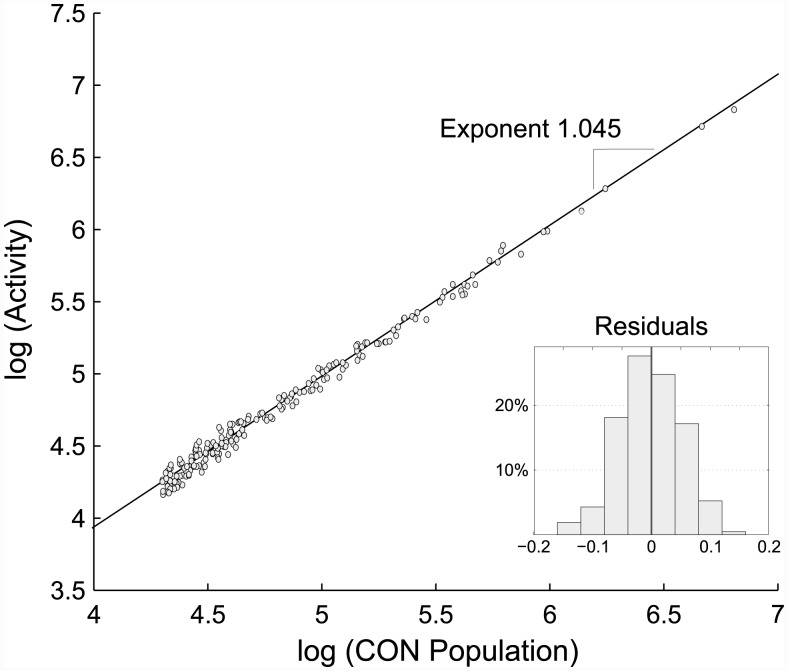
Superlinear scaling of total spending activity with city size for the Conurbation level. Total spending activity is defined as the cumulative number of transactions made by city residents. Scaling exponent: 1.045, confidence interval: [1.03,1.06], p-value: 4 ⋅ 10^−205^, *R*^2^ = 99.0%.

**Fig 5:**
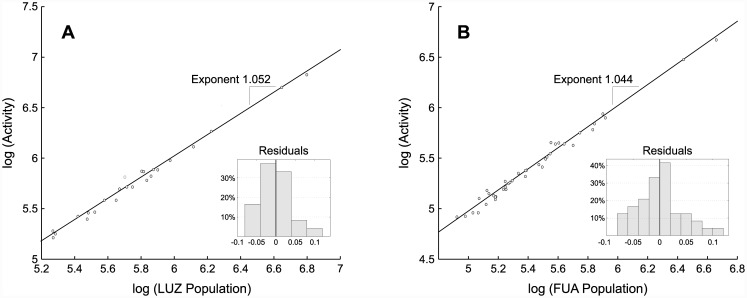
Superlinear scaling of total spending activity with city size. A: For the level of Large Urban Zones. Scaling exponent: 1.052, CI: [1.00,1.10], p-value: 5 ⋅ 10^−23^, *R*^2^ = 98.90%. B: For the level of Functional Urban Areas. Scaling exponent: 1.044, CI: [1.00,1.08], p-value: 1 ⋅ 10^−37^, *R*^2^ = 98.71%. Total spending activity is defined as the cumulative number of transactions made by city residents.

In Fig 4, the total activity of each Conurbation is plotted against its size at the log-log scale. We observe a superlinear scaling with the exponent of 1.045. Statistical significance of the trend is further validated by considering the confidence interval for the exponent. Fig 5 confirms this finding for the two other levels of city definition (LUZ and FUA). Importantly, the exponents for all city levels are approximately the same (around 1.05), indicating that the uncovered scaling is a distinctive feature of urban areas, regardless of the chosen city boundary.

As we have just demonstrated, the bank card spending of individuals follows the same pattern that has been found for other socioeconomic parameters—they are boosted up in bigger cities. However, the exponent we obtained is lower than those obtained for other aspects of human activity (e.g. human communication scales with the exponent of 1.12 [39]). One of the reasons might be that the considered total spending actually includes a broad range of purchases of different goods and services. Some of them are common needs, which exist regardless of the place of residence (buying food for instance), and their volume does not depend on city size that much, while there are also more specific purchases that largely depend on the available options and might even be -at least partially—motivated by those. The hierarchy of purchases and its triangular composition was explored by [26], who suggested that its specific structure may indeed be place-dependent.

With the data at our disposal, we are also able to distinguish different target categories of businesses. Table 1 gives the scaling exponents for a few major business categories. To confirm the validity of the trends, we also provide confidence intervals and p-values. The fraction of activity represented by the corresponding business category is in the last column. As can be seen, entertaining activities like traveling, going out for a drink, diner or a party are strongly boosted by city size, with a scaling exponent over 1.1. In bigger cities, people seem to engage more easily in social activities, which confirms the suggestion of [39]. Similarly, categories such as wellness, beauty, and fashion also possess higher-than-average, statistically significant exponents. On the contrary, fundamental needs are less impacted by city size, with exponent values almost equal to 1. Living in a big or a small town does not affect one’s attendance to grocery stores, supermarkets or gas stations. And since those activities cover 40% of the total spending, they considerably lower the overall scaling exponent.

**Table 1:** Scaling of customers’ activity with CON size for different business categories.

Business category	Exponent	Confidence intervals	p-value	Fraction of activity
Everyone	1.045	[1.03,1.06]	4e-205%	100%
Bars, restaurants, and clubs	1.170	[1.13, 1.21]	1e-6%	6.95%
Travels	1.118	[1.070,1.167]	2.8e-6%	6.44%
Health institutions	1.075	[1.042, 1.107]	1.0e-5%	4.78%
Entertainment	1.145	[1.085, 1.204]	2.9e-6%	0.45%
Gas	1.004	[0.974,1.032]	76.0%	10.01%
Supermarkets	1.027	[0.990,1.055]	5.84%	29.47%
Wellness, beauty and fashion	1.082	[1.052, 1.112]	1.8e-7%	16.92%
Others	1.070	[1.023, 1.116]	0.35%	24.97%

The other spending parameters considered in this paper demonstrate diverse behaviors in terms of scaling. Let us go over a few quantities (all others, together with graphs for diversity, can be found in S1 Table and S2 Fig). The average amount per transaction seems to be generally independent from city size. Conversely, a statistically significant scaling trend appears for individual average spending diversity, suggesting that customers from larger cities have a slightly broader variety of purchases. The exponent of 0.08 together with a p-value of 6 ⋅ 10^−22^% for Conurbations proves the increasing trend for the average diversity with nearly 100% confidence. The trend is also statistically significant for LUZ and FUA. In case of mobility, the patterns are mixed. The most meaningful relation is observed for the CON level: local mobility exhibits a downward trend, with a scaling exponent of -0.076, while distant travels show a positive scaling with a noticeable exponent of 0.130. It appears that in larger cities, people are able to satisfy their shopping needs closer to their home, thus sparing them from longer journeys to perform local transactions. The uncovered trend for distant mobility (strongly increasing with population) indicates that inhabitants of large cities explore the country on a wider scale. Nevertheless, mobility trends strongly depend on the city definition level. The patterns at the level of LUZ are quite different from those at a core city scale.

## Classification of Spanish cities beyond the impact of demography and scale

Scaling laws described in the previous section explain how the economic behavior of city residents is expected to change with city size. However, the actual values of spending parameters deviate from the estimations. For example, as can be seen from S1 Table, even when the trends are statistically significant, the portion of the observed variations they are able to explain (*R*^2^) sometimes happens to be as low as 20%. Values higher than the corresponding trend can be treated as overperformance of the city. On the contrary, values below the trend may be interpreted as underperformance. Similarly to [41], we quantify those deviations using log-scale residuals (i.e., the difference between the decimal logarithm of the actual city characteristic and the decimal logarithm of the corresponding trend estimation). These residuals are computed in relation to size-specific estimates and can therefore be used for a qualitative comparison of different cities, regardless of their population. Since the five urban parameters were normalized beforehand for age and gender variability, the residuals are also free from the impact of demography. Altogether, this approach allows the definition of a novel classification of Spanish cities, revealing the impact of local circumstances on residents’ spending behavior.

Residuals of the five urban parameters represent the distinctive signature of a city. We consider each city definition separately, which results in three separate sets of city signatures. In order to bring the residuals of different parameters on a common scale, we normalize them by their standard deviation (the mean being always zero by definition of a trend). Similarity between the signatures of cities within a given level is assessed with the k-means clustering algorithm [48]. In order to stabilize the separation of clusters, we apply the majority voting across several dozens of iterations. According to the silhouette metric [49], the most optimal solution divides cities into three clusters at all levels but LUZs (see S2 Text and S3 Fig for a detailed description of the metric and computed values). However, at all levels, the division into three clusters forms a pattern that is meaningful for the qualitative interpretation of results. Interestingly, we also observe a largely consistent hierarchy between the two- and three-cluster cases, quantified as 90% agreement for CON, 85% for FUA, and 100% for LUZ (given as proportion of cities remaining in the same cluster, under the assumption that one of the two clusters remains and the other one splits in two). Such properties were not observed with larger numbers of clusters. Therefore, the classification into three categories of cities is selected as the basic one for the next results presentation.

### Spending profiles of the received categories of Spanish cities

**Fig 6:**
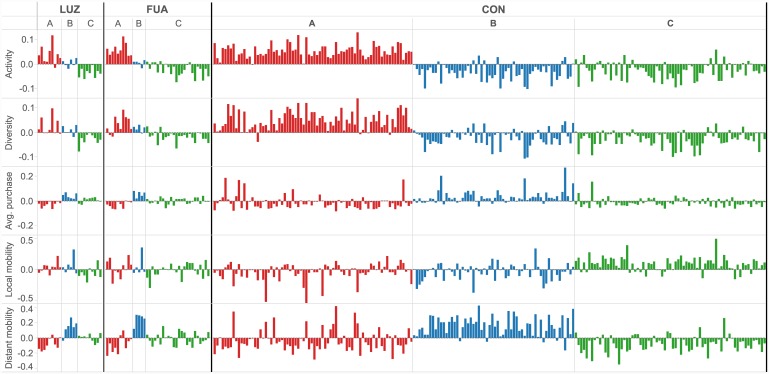
Deviations of spending parameters from their respective scaling trends with city size, for the cities defined at the level Large Urban Zones (LUZ), Functional Urban Areas (FUA), and Conurbations (CON). Colors indicate three clusters of cities obtained based on the k-mean clustering, in accordance with Fig 7.

Each of the received city clusters can be characterized with a distinct profile of residents’ spending behavior. Differences between particular profiles are well recognizable from the deviations of particular bank card usage parameters (Fig 6). Major distinctions are provided by the combination of spending activity and diversity, as well as distant mobility. The first two parameters, which are in any case correlated as higher number of transactions implies higher diversity, explain the separation of cluster A (red). The variations of distant mobility justify the additional split of clusters B (blue) and C (green). More importantly, those distinctions are consistent across the city levels.

**Fig 7:**
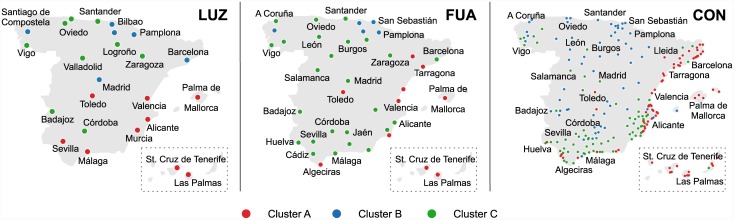
Classification of Spanish cities into three categories based on the spending behavior of their residents. Classification was performed separately for the three different city definition levels–Large Urban Zones (LUZ), Functional Urban Areas (FUA), and Conurbations (CON). Clustering into two categories can be, to large extent, recreated by merging clusters B and C into one (the consistency between the two- and three-cluster cases was quantified as high as 100% for LUZ, 98% for FUA, and 97% for CON).

Received clusters can be further interpreted based on their spatial alignment (Fig 7), which exhibits a high robustness across the three city definitions. The variations can be attributed, to a large degree, to the changes of sample size and spatial scale (e.g., different mobility patterns within LUZ and CON). In general, a good agreement across city levels is observed and can serve as additional evidence for the consistency and stability of the approach. Below, we present a detailed interpretation of the three received city clusters.

The most distinctive city category is the red cluster, covering cities located along the most visited part of the Mediterranean coast and on the islands. This pattern clearly refers to the most touristic parts of Spain, which is further supported by the incorporation of Toledo, a World Heritage Site by UNESCO. The appearance of this “touristic” cluster is interesting, as our procedure relies exclusively on the economic activity of city residents—indicating that inhabitants of this type of cities have a distinctive economic behavior. The spending profile of the red cluster is characterized by an intensified spending activity and diversity, which are accompanied by an underperformance of the average purchase. This indicates that residents use their bank cards more often than for the occasional shopping, covering all types of small, everyday purchases. Negative deviations recorded for the distant mobility parameter are well understandable from the perspective of the geographic distribution of cities, especially for the isolated island cities.

The core of the blue cluster includes Santiago de Compostela and cities from the Basque and Navarra regions. In case of LUZ, it also covers Madrid and Barcelona. In case of CON, it expands into a much wider area in the north as well as part of the Spanish interior. Cities grouped within this cluster exhibit negative, or close to average, residuals for the activity and diversity parameters, while the residuals for the average purchase tend to be positive. The latter goes in line with the economic profile of the core cities in the blue cluster—wealthy industrial cities in the northern part of Spain, such as Bilbao or Pamplona. Higher purchase power may also be the reason for the distant transaction values, which are much higher than expected from the scaling trend. However, this larger spending potential is combined with a decreased intensity of card usage. This points to a conclusion opposite to that of the red cluster—bank cards are used for substantial amounts of money but quite rarely.

Green cluster concentrates cities of the Spanish interior and the remaining ones from the north. Similar to the blue cluster, it is characterized by negative residuals for activity and diversity. Deviations of the remaining three parameters are of a different type. The average purchase values are close to the baseline provided by the general trend. At the same time, distant mobility mainly records negative deviations, especially at the level of CON. Accompanied by a larger local mobility for CON, this observation suggests an economic activity of residents concentrated around their cities, which are satisfying most of their needs. It agrees well with the fact that the green cluster consists of many service-oriented cities, serving as administrative capitals for big territories, such as Valladolid and Zaragoza (at the levels of LUZ and FUA).

### Socioeconomic profiles of the clusters

Three Spanish cities classifications have been built solely based on the individual economic activity of their residents. In this section, we examine how well these clusters agree with standard socioeconomic statistics. We picked three major urban indicators, available for LUZ and FUA thanks the Urban Audit Survey [42] and the Spanish National Statistics Institute (INE) [45]. These are Gross Domestic Product (GDP, estimated based on the province quantities), unemployment (in absolute numbers), and the average disposable annual household income (further referred to as income). All statistics reflect the state in 2011, with the exception of the unemployment figures for LUZ, available only for 2009. Data at the CON level were not available to the authors. Additionally, we compare LUZ spendings with the Household Budget Survey of INE from 2011 (Total expenditure, further referred to as households’ expenditure), available only at the coarse spatial resolution of Autonomous Communities.

As already mentioned, socioeconomic indicators are proven to scale superlinearly with city size [37, 40]. We confirmed that this property holds also for our socioeconomic data, with the exception of the income at the level of FUA, where the relation with city population is rather linear. Therefore, we construct socioeconomic metrics in the same way as our five spending parameters, based on the log deviations from the scaling estimates. This way, the metrics indicate if city scores are better or worse than the expected performance of a city of that size. For the households’ expenditure, residuals obtained for the Autonomous Communities were assigned to corresponding LUZ. Residuals for all socioeconomic indicators are available at http://senseable.mit.edu/BBVA/LUZ_CON_FUA_spendingSignatures.xlsx.

Thorough linear correlation can only be observed for average purchase amount. It is particularly strong for the households’ expenditure at the LUZ level (*R*^2^ = 0.78), showing a big opportunity for bank card data to extend this standard statistics with a better spatial and, potentially, temporal resolution, and with a much larger sample size (4.5 million of BBVA customers vs. 24,000 of surveyed households). As far as other parameters are concerned, the average purchase value is negatively correlated with unemployment (*R*^2^ = 0.82 / LUZ and 0.65 / FUA) and positively correlated with income (*R*^2^ = 0.49 / LUZ and 0.37 / FUA) and GDP (*R*^2^ = 0.55 / LUZ 0.34 / FUA). Weaker, though still visible, correlations can also be noted between the aforementioned indicators and distant mobility. The other spending parameters do not show a direct linear correlation with socioeconomic statistics.

**Fig 8:**
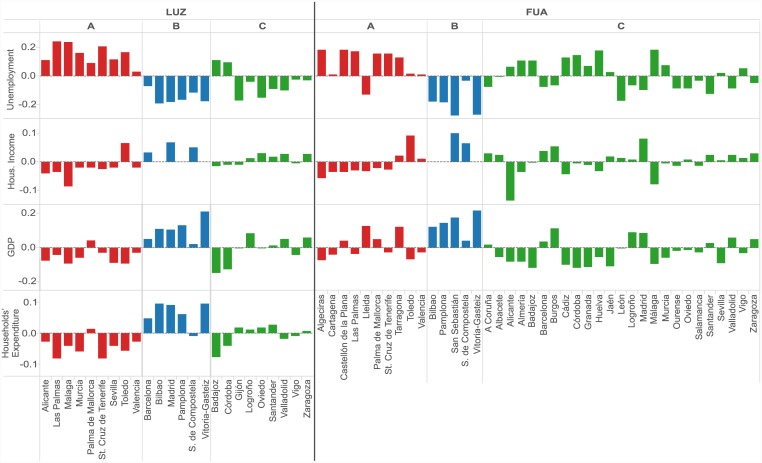
Deviations of standard socioeconomic statistics within the three clusters of Spanish cities at the level of LUZ, and FUA. Unemployment, Disposal Annual Income of Households, and Gross Domestic Product were available for both city levels. Total Expenditure of Households, from the Household Budget Survey, was available only for the Autonomous Communities and reassigned to the corresponding LUZ. The data comes for the Urban Audit survey of Eurostat [42] and the Spanish National Statistics Institute [45]. Socioeconomic parameters were quantified as log residuals from their respective scaling trends with city size. Colors correspond to the three clusters of cities obtained based on the spending behavior of city residents, as presented in Fig 7.

Nevertheless, interesting patterns appear when crossing the deviations of socioeconomic metrics with the three received city clusters (Fig 8). The most distinctive observation concerns cluster B (blue), where the majority of cities exhibit high positive residuals for income, GDP and households’ expenditure (with the exception of Santiago de Compostela, values of which is very close to the trend line) and negative residuals for unemployment. It confirms a good socioeconomic condition in the cities of the blue cluster and agrees well with their spending profile—higher than expected average purchase values and distant mobility. The situation seems to be just the opposite for cluster A (red). A majority of cities record high residuals for unemployment and underperformance in terms of households’ expenditure, income, and GDP, which may indicate both social and economic problems. Individually, these go hand in hand with the decreased average purchase values and distant mobility but still allow a high level of general spending activity. Interpretation for cluster C (green), characterized by a mix of residuals’ values, does not seem to be as straightforward as for the previous ones; however, we observe a slight tendency to underperform in GDP, while the income and expenditure residuals stay close to the trend. Together with the previously reported low levels of spending activity and diversity, this suggests that although the average income of households is at the expected level, it is spent for a rather limited number of purchases, and does not contribute to the improvement of the economic well-being of cities. Observations on the correspondence of the three city clusters and socioeconomic statistics are generally consistent across city levels (with the exception of households’ expenditure, complete data not being available), which supports their validity.

### Attractiveness of the clusters to foreign visitors

**Fig 9:**
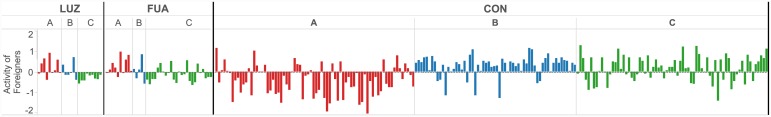
Correspondence of the received clusters of Spanish cities with the attractiveness of these cities. Attractiveness is calculated based on the spending activity of foreign visitors. Presented deviations are quantified as log residuals from their respective scaling trends with city size at the levels of LUZ, FUA and CON. Colors correspond to the three clusters of cities obtained based on the spending behavior of city residents, as presented in Fig 7.

We have already mentioned that, even though clustering was performed solely based on the activity of city residents without taking visitors into account, many touristic parts of Spain are gathered in cluster A. To explore this observation in a more quantitative manner, we compared our clusters with Spanish cities attractiveness to foreign visitors. As proposed in [29, 30], city attractiveness can be captured using the spending activity of its foreign visitors. This activity exhibits a strong superlinear scaling with city size, with an exponent of around 1.5 [30]. Therefore, residuals from the scaling trends at each city level indicate how much a city is attractive to foreigners relative to the average expectation of any city of the same size. That measure, constructed in the same manner that the five spending parameters for residents, can be put in relation with the distribution of cities in clusters. We perform that comparison using data containing bank card purchases of foreigners visiting Spain, its results can be found in Fig 9. Most of the cities gathered in cluster A are indeed characterized by a foreigners spending activity way above the trend line, while a majority of the cities from the remaining clusters tend to underperform on this measure. The pattern is especially evident at the level of CON, but to a minor extent, it is also visible for LUZ and FUA. In general, the correspondence of foreign attractiveness and spending habits of city residents is an interesting observation, which shows the great extent to which the touristic profile of a city affects the individual life of its inhabitants. At the same time, it also puts the proposed classification in a much broader perspective going beyond the original idea of the impact of cities on the economic behavior of their residents.

## Conclusions

In the present study we explored individual economic behavior of urban residents by means of their bank card transactions, quantified through five parameters: customer activity, average value of transactions, spending diversity, local mobility, and distant mobility. On a general level, we confirmed the substantial impact of age, gender and place of residence on human spending behavior. In particular, we found out that all of the above parameters scale quantitatively with city population, and thus form a good basis for a bottom-up economic descriptors of urban well-being, which was confirmed with the creation of a new and meaningful clustering of Spanish cities.

Exploring the connection between individual economic activity and demographics, we extended the findings of [28], showing that age and gender have a major impact on all of the bank card spending parameters. Consistent trends were obtained when correlating them with age, and the curves for genders were different from one another, similar in shape but not in absolute terms. For instance, the average purchase amount demonstrates a surprisingly steady linear increase with customer age. This phenomenon can be interpreted as a general tendency to group purchases when growing old. Also, the spending diversity peaks around 30 years old, a fact consistent with the average age of first childbirth, and then starts to decrease. The confirmed impact of customers’ demographics on their bank card activity implies the necessity to compensate for such factors while building and comparing aggregated patterns—in our case, the spending profiles of different urban areas.

Next, we found that the size of the city of residence has a noticeable impact on all the characteristics of individual spending behavior, except for the average amount of purchases, in a way that can often be described as a statistically significant power law scaling. The overall spending activity scales superlinearly with city size—a fact that goes in line with the previous findings regarding other socioeconomic urban quantities. At the same time, individual spending in different types of businesses shows a substantially different scaling behavior, which hints to a noticeable shift in the categories of customer activity within cities of different scales.

However, each city posseses its own unique characteristics in terms of spending parameters, different from the general superlinear scaling. We demonstrated that the deviations from the trends’ baselines can be regarded as distinct signatures of cities, forming a solid basis for a scale-free comparison and classification of urban areas. The approach was tested on Spanish cities defined at three different scales—Large Urban Zones, Functional Urban Areas and Conurbations. This purely data-driven classification, independent from any spatial or topological considerations, revealed three meaningful categories of Spanish cities. The categories have shown to be distinct in terms of spending patterns of their residents, geographic alignment and the standard socioeconomic statistics (derived from the external metrics of GDP, unemployment and income). At the level of LUZ, they also corresponded well with the Spanish Household Budget Survey, available at the far coarser spatial resolution of Autonomous Communities. In particular, the average value of bank transactions exhibited a high correlation with households’ expenditures, 78%, showing the potential of bank card data for extending some of the standard statistics with a better spatial and, potentially, temporal resolution. Furthermore, the obtained clustering results were found to capture meaningful economic patterns beyond the scope of the considered data—certain categories, while being derived solely based on the behavior of city residents, corresponded well with the attractiveness of those cities to foreign visitors.

As a final remark, we should point out that the proposed classification remained, to a certain extent, stable for the different city definitions, which constitutes additional evidence of its robustness. Moreover, although in the paper the approach is applied to Spain, we believe it to be widely applicable to any other country, provided that the appropriate data is available.

## Supporting Information

S1 Text Demographic normalization. (PDF)

S2 Text Validation of the clustering scheme. (PDF)

S1 Table Scaling of the five characteristics of individual spending behavior with city size. (PDF)

S1 Fig Impact of age and gender on the total amount of money spent by BBVA customers in 2011. (EPS)

S2 Fig Scaling of spending diversity with city size. As mentioned in the main text, the spending diversity (measured using the HH Index) exhibits a small but consistent scaling with city size for the three definitions considered: Large Urban Zones (LUZ), Functional Urban Areas (FUA), and Conurbations (CON). LUZ exponent: 0.057, CI: [0.02,0.1], p-value: 0.8%, *R*^2^ = 27.5%. FUA exponent: 0.064, CI: [0.03,0.09], p-value: 9*e*-5%, *R*^2^ = 33.3%. CON exponent: 0.08, CI: [0.7,0.1], p-value: 6*e*-22%, *R*^2^ = 36.2%.
(EPS)

S3 Fig Values of the silhouette metric for different k-mean clustering schemes. We varied *k* between 2 and 10, separately for the three levels of city definition: Large Urban Zones (LUZ), Functional Urban Areas (FUA), and Conurbations (CON). Higher values indicate a better fit of data points to the clusters they were assigned (more appropriate clustering approach).
(EPS)

## References

[1] Girardin F, Calabrese F, Fiore FD, Ratti C, Blat J. Digital Footprinting: Uncovering Tourists with User-Generated content. Pervasive Computing, IEEE. 2008;7: 5276 10.1109/MPRV.2008.71

[2] González MC, Hidalgo CA, Barabási AL. Understanding individual human mobility patterns. Nature. 2008;453: 779–782. 10.1038/nature06958 18528393

[3] Quercia D, Lathia N, Calabrese F, Di Lorenzo G, Crowcroft J. Recommending Social Events from Mobile Phone Location Data. In: Data Mining (ICDM), 2010 IEEE 10th International Conference on; 2010. pp. 971–976.

[4] Sobolevsky S, Szell M, Campari R, Couronné T, Smoreda Z, Ratti C. Delineating Geographical Regions with Networks of Human Interactions in an Extensive Set of Countries. PloS ONE. 2013;8(12): e81707 10.1371/journal.pone.0081707 24367490 PMC3867326

[5] Amini A, Kung K, Kang C, Sobolevsky S, Ratti C. The impact of social segregation on human mobility in developing and industrialized regions. EPJ Data Science. 2014;3(1): 6 10.1140/epjds31

[6] Santi P, Resta G, Szell M, Sobolevsky S, Strogatz SH, Ratti C. Quantifying the benefits of vehicle pooling with shareability networks. Proceedings of the National Academy of Sciences. 2014;111(37): 13290–13294. 10.1073/pnas.1403657111 PMC4169909 25197046

[7] Szell M, Grauwin S, Ratti C. Contraction of Online Response to Major Events. PloS ONE. 2014;9(2): e89052 10.1371/journal.pone.0089052 24586499 PMC3935844

[8] Frank MR, Mitchell L, Dodds PS, Danforth CM. Happiness and the Patterns of Life: A Study of Geolocated Tweets. Scientific Reports. 2013;3: 2625 10.1038/srep02625 24026340 PMC6505625

[9] Hawelka B, Sitko I, Beinat E, Sobolevsky S, Kazakopoulos P, Ratti C. Geo-located Twitter as proxy for global mobility pattern. Cartography and Geographic Information Science. 2014;41(3): 260–271. 10.1080/15230406.2014.890072 27019645 PMC4786829

[10] Paldino S, Bojic I, Sobolevsky S, Ratti C, González MC. Urban Magnetism through the Lens of Geo-tagged Photography; 2015. EPJ Data Science. 2015;4(1): 17 10.1140/epjds/s13688-015-0043-327990325

[11] Bagchi M, White PR. The potential of public transport smart card data. Transport Policy. 2005;12(5): 464–474.

[12] Louail T, Lenormand M, Cantu-Ros OG, Picornell M, Herranz R, Frias-Martinez E, et al From mobile phone data to the spatial structure of cities. Scientific Reports. 2014;4: 5276 10.1038/srep05276 24923248 PMC4055889

[13] Ratti C, Sobolevsky S, Calabrese F, Andris C, Reades J, Martino M, et al Redrawing the map of Great Britain from a network of human interactions. PLoS ONE. 2010; 5(12): e14248 10.1371/journal.pone.0014248 21170390 PMC2999538

[14] Grauwin S, Sobolevsky S, Moritz S, Gódor I, Ratti C. Towards a Comparative Science of Cities: Using Mobile Traffic Records in New York, London, and Hong Kong. Computational Approaches for Urban Environments. 2015;13: 363–387. 10.1007/978-3-319-11469-9_15

[15] Pei T, Sobolevsky S, Ratti C, Shaw SL, Li T, Zhou C. A new insight into land use classification based on aggregated mobile phone data. International Journal of Geographical Information Science. 2014; 28(9): 1988–2007. 10.1080/13658816.2014.913794

[16] Noulas A, Scellato S, Lambiotte R, Pontil M, Mascolo C. A Tale of Many Cities: Universal Patterns in Human Urban Mobility. PLoS ONE. 2012;7(5): e37027 10.1371/journal.pone.0037027 22666339 PMC3362592

[17] Kung K, Greco K, Sobolevsky S, Ratti C. Exploring Universal Patterns in Human Home-Work Commuting from Mobile Phone Data. PLoS ONE. 2014;9(6): e96180 10.1371/journal.pone.0096180 24933264 PMC4059629

[18] Lathia N, Quercia D, Crowcroft J. The Hidden Image of the City: Sensing Community Well-Being from Urban Mobility. In: Kay J, Lukowicz P, Tokuda H, Olivier P, Krüger A, editors. Pervasive Computing. vol. 7319 of Lecture Notes in Computer Science; 2012. pp. 91–98.

[19] Arribas-Bell D, Kourtit K, Nijkamp P. Benchmarking of world cities through Self-Organizing Maps Cities. 2013;31: 248–257.

[20] Lloyd R, Jennings D. Shopping Behavior and Income: Comparisons in an Urban Environment. Economic Geography. 1978;54(2): 157–167. 10.2307/142850

[21] Childers TL, Car CL, Peck J, Carson S. Hedonic and utilitarian motivations for online retail shopping behavior. Journal of Retailing. 2001;77(4): 511–535.

[22] Dholakia RR. Going shopping: key determinants of shopping behaviors and motivations. International Journal of Retail & Distribution Management. 1999;27(4): 154–165. 10.1108/09590559910268499

[23] Chan PK, Fan W, Prodromidis AL, Stolfo SJ. Distributed data mining in credit card fraud detection. Intelligent Systems and their Applications (IEEE). 1999;14(3): 67–74. 10.1109/5254.809570

[24] Rysman M. An Empirical Analysis of Payment Card Usage. The Journal of Industrial Economics. 2007;55(1): 1–36. 10.1111/j.1467-6451.2007.00301.x

[25] Krumme C, Llorente A, Cebrian M, Pentland A, Moro E. The predictability of consumer visitation patterns. Scientific Reports. 2013;3: 1645 10.1038/srep01645 23598917 PMC3629735

[26] Pennacchioli D, Coscia M, Rinzivillo S, Giannotti F, Pedreschi D. The retail market as complex system. EPJ Data Science. 2014;3(33): 1–27.

[27] Sobolevsky S, Sitko I, Tachet des Combes R, Hawelka B, Murillo Arias J, Ratti C. Money on the Move: Big Data of Bank Card Transactions as the New Proxy for Human Mobility Patterns and Regional Delineation. The Case of Residents and Foreign Visitors in Spain. In: Big Data (BigData Congress), 2014 IEEE International Congress on, Jun 27–Jul 2, Anchorage, AK; 2014. p. 136–143.

[28] Lenormand M, Louail T, Cantu-Ros OG, Picornell M, Herranz R, Murillo Arias J, et al Influence of sociodemographics on human mobility; 2015. Scientific Reports. 2015;5: 10075 10.1038/srep10075 25993055 PMC4438721

[29] Sobolevsky S, Sitko I, Grauwin S, Tachet des Combes R, Hawelka B, Murillo Arias J, et al. Mining Urban Performance: Scale-Independent Classification of Cities based on Individual Economic Transactions. In: Big Data Science and Computing, 2014 ASE International Conference on, May 27–31, Stanford University; 2014. Available: http://www.ase360.org/bitstream/handle/123456789/48/Poster71.pdf?sequence=3&isAllowed=y.

[30] Sobolevsky S, Bojic I, Belyi A, Sitko I, Hawelka B, Murillo Arias J, et al. Scaling of city attractiveness for foreign visitors through big data of human economical and social media activity; 2015. Preprtint. Available: arXiv:1504.06003. Accessed 23 April 2015.

[31] Bhantagar A, Misra S, Rao HR. On Risk, Convenience, and Internet Shopping Behavior. Communications of the ACM. 2000;43(11): 98–105. 10.1145/353360.353371

[32] Hui TK, Wan D. Factors affecting Internet shopping behaviour in Singapore: gender and educational issues. International Journal of Consumer Studies. 2007;31(3): 310–316. 10.1111/j.1470-6431.2006.00554.x

[33] Hayhoe CR, Leach LJ, Turner PR, Bruin MJ, Lawrence FC. Differences in Spending Habits and Credit Use of College Students. Journal of Consumer Affairs. 2008;34(1): 113–133. 10.1111/j.1745-6606.2000.tb00087.x

[34] Borzekowski R, Kiser EK, Ahmed S. Consumers’ Use of Debit Cards: Patterns, Preferences, and Price Response. Journal of Money, Credit and Banking. 2008;40(1): 149–172. 10.1111/j.1538-4616.2008.00107.x

[35] Bounie D, Francois A. Cash, Check or Bank Card? The Effects of Transaction Characteristics on the Use of Payment Instruments. SSRN Scholarly Paper. 2006;(ID 89179).

[36] Batty M. The Size, Scale, and Shape of Cities. Science. 2008;319(5864): 769–771. 10.1126/science.1151419 18258906

[37] Bettencourt LM, Lobo J, Helbing D, Kühnert C, West GB. Growth, innovation, scaling, and the pace of life in cities. Proceedings of the National Academy of Sciences. 2007;104(17): 7301–7306. 10.1073/pnas.0610172104 PMC1852329 17438298

[38] Brockmann D, Hufnagel L, Geisel T. The scaling laws of human travel. Nature. 2006;439(7075): 462–465. 10.1038/nature04292 16437114

[39] Schläpfer M, Bettencourt L, Grauwin S, Raschke M, Claxton R, Smoreda Z, et al The Scaling of Human Interactions with City Size. Journal of the Royal Society Interface. 2014;11(98): 20130789 10.1098/rsif.2013.0789 PMC4233681 24990287

[40] Bettencourt LM. The Origins of Scaling in Cities. Science. 2013;340(6139): 1438–1441. 10.1126/science.1235823 23788793

[41] Bettencourt LM, Lobo J, Strumsky D, West GB. Urban Scaling and Its Deviations: Revealing the Structure of Wealth, Innovation and Crime across Cities. PLoS ONE. 2010;5(11): e13541 10.1371/journal.pone.0013541 21085659 PMC2978092

[42] Eurostat. Urban Audit. Available: http://ec.europa.eu/eurostat/web/cities. Accessed 2 October 2014.

[43] AUDES—Aŕeas Urbanas de Espanã. Available: alarcos.esi.uclm.es/per/fruiz/audes. Accessed 24 October 2014.

[44] ESPON project 1.4.3: Study on Urban Functions: Final Report. 2007. Available: http://www.espon.eu/main/Menu_Projects/Menu_ESPON2006Projects/Menu_StudiesScientificSupportProjects/urbanfunctions.html.

[45] Instituto Nacional Estadística. Available: www.ine.es Accessed 26 October 2014.

[46] World Factbook (The). Mother’s mean age at first birth. Available: https://www.cia.gov/library/publications/the-world-factbook/fields/2256.html. Accessed 7 November 2014.

[47] Arcaute E, Hatna E, Ferguson P, Youn H, Johansson A, Batty M. Constructing cities, deconstructing scaling laws; 2014. Preprint. Available: arXiv:13011674. Accessed 15 December 2014. 10.1098/rsif.2014.0745 PMC4277074 25411405

[48] MacQueen J. Some methods for classification and analysis of multivariate observations. Proceedings of the Fifth Berkeley Symposium on Mathematical Statistics and Probability. 1967;1: Statistics: 281–297.

[49] Rousseeuw P. Silhouettes: A graphical aid to the interpretation and validation of cluster analysis. Journal of Computational and Applied Mathematics. 1987;20: 53–65.

