# Supplementary material for: Cities through the Prism of People’s Spending Behavior
Source: PLoS One. 2016 Feb 5;11(2):e0146291. doi: 10.1371/journal.pone.0146291 (PMC4743849; doi:10.1371/journal.pone.0146291)
Supplement: S1 Text — (PDF) [file pone.0146291.s001.pdf]

S1 Text. Demographic normalization

Let us describe the normalization procedure used to account for demographic discrepancies between cities. The idea is to compare the observed value of a given parameter with its theoretical expected one (computed using the city demographic profile). Let $(p_c)_{c \in C}$ be the measured parameter where C denotes the entire set of customers, C_X the subset containing only customers from city X and $C_{g,a}$ the customers of gender g and age a. The average quantity for a given gender g and age a and for a given city X are

$$Q_{g,a} = \frac{\sum\limits_{c \in C_{g,a}} p_c}{|C_{g,a}|} \qquad Q_X = \frac{\sum\limits_{c \in C_X} p_c}{|C_X|}$$

The expected value of the parameter based on the demography of city X is

$$E_X = \frac{\sum\limits_{g,a} |C_X \cap C_{g,a}| Q_{g,a}}{|C_X|}$$

In the end, the normalized value used as a measure of city X economic behavior is $\frac{Q_X}{E_X}$
