# Supplementary material for: Cities through the Prism of People’s Spending Behavior
Source: PLoS One. 2016 Feb 5;11(2):e0146291. doi: 10.1371/journal.pone.0146291 (PMC4743849; doi:10.1371/journal.pone.0146291)
Supplement: S2 Text — (PDF) [file pone.0146291.s002.pdf]

S2 Text. Validation of the clustering scheme

Similarity between the signatures of cities within each definition level i.e., CONs, FUAs and LUZs, was assessed with the k-means clustering algorithm [1]. In order to select the optimal number of clusters, we validated different approaches with the silhouette metric *s(i)* [2], with $k \in [2;10]$. Silhouette aims to reflect how well each object fits to its cluster based on the comparison of an object dissimilarity (in our case, the Euclidean distance) to the points grouped in the same cluster and to the points grouped within the next best fitting cluster. It is computed according to the equation:

$$s(i) = \frac{b(i) - a(i)}{max\{a(i), b(i)\}},$$

where *a(i)* is the average dissimilarity of a city i to the other cities assigned to the same cluster, and *b(i)* is the average dissimilarity of a city i to its next best fitting cluster. Silhouette varies in the range of [-1;1]. Positive values indicate a good match with the own cluster (small *a(i)*) and a bad match with the neighboring cluster (high *b(i)*). On the contrary, negative values indicate that a data points is more similar to the neighboring cluster, while values around 0 imply that a point is on the edge of two clusters.

We compared the average values of *s(i)* across the clustering schemes with different k, assuming that the highest values indicate the optimal split of cities. Received values are presented in Figure S3. We observe that the silhouette metric peaks at $k = 3$ for the levels of CONs and FUAs, and $k = 5$ for LUZs. However, at all levels, the division into three clusters introduces a pattern that is meaningful for the qualitative interpretation of results. Such property is not observed with the further increase of the number of clusters. Therefore, and for the sake of consistency, the classification into three categories of cities is retained as the basic one for the the presentation of paper results. Additionally, we validated selected algorithm i.e., k-means, against its common variation i.e., k-medoids [3]. As the latter one resulted in lower silhouette values for all tested k, we retained the k-means approach.

References

1. MacQueen J. Some methods for classification and analysis of multivariate observations. Proceedings of the Fifth Berkeley Symposium on Mathematical Statistics and Probability. 1967;1: Statistics: 281–297.

2. Rousseeuw P. Silhouettes: A graphical aid to the interpretation and validation of cluster analysis. Journal of Computational and Applied Mathematics. 1987;20: 53–65.

3. Kaufman L, Rousseeuw P. Clustering by means of medoids. In: Dodge Y, editor. Statistical Data Analysis Based on the L1 Norm and Related Methods. North-Holland; 1987. p. 405–416.
