# Supplementary material for: Cities through the Prism of People’s Spending Behavior
Source: PLoS One. 2016 Feb 5;11(2):e0146291. doi: 10.1371/journal.pone.0146291 (PMC4743849; doi:10.1371/journal.pone.0146291)
Supplement: S1 Table — (PDF) [file pone.0146291.s003.pdf]

S1 Table. Scaling of the five characteristics of individual spending behavior with city size.

Parameter	City definition	Exponen	Confidence intervals	p-value	R^2
Activity	LUZ	1.052	[1.0,1.1]	5e-23 %	98.90%
	FUA	1.044	[1.0,1.08]	1e-37 %	98.71%
	CON	1.045	[1.03,1.06]	4e-205 %	99.0%
Avg. amount	LUZ	-0.006	[-0.05,0.03]	75.3 %	0.46%
	FUA	0.005	[-0.03,0.04]	78.2 %	0.2 %
	CON	0.01	[-0.005,0.03]	18.9 %	0.8%
Diversity Herf.	LUZ	0.057	[0.016,0.099]	0.85 %	27.51%
	FUA	0.064	[0.034,0.093]	9e-5 %	33.29%
	CON	0.080	[0.066,0.095]	6e-22 %	36.16%
Distant mob.	LUZ	-0.128	[-0.26,0.0]	5.76 %	15.4%
	FUA	-0.007	[-0.13,0.11]	89.2%	5e-4%
	CON	0.130	[0.08,0.18]	8e-8 %	13.0%
Local mob.	LUZ	-0.059	[-0.19,0.07]	36.1 %	3.8%
	FUA	-0.026	[-0.14,0.09]	63.9 %	0.6%
	CON	-0.076	[-0.12,-0.03]	0.02 %	6.38%
